# Is there an association between glenoid parameters and rotator cuff tears and the influence of gender: A retrospective study on a Middle Eastern population

**DOI:** 10.1016/j.ijscr.2020.02.035

**Published:** 2020-02-21

**Authors:** Joseph Maalouly, Antonios Tawk, Dany Aouad, Hicham Abdel Nour, Elias Saidy, Ghadi Abboud, Georges El Rassi

**Affiliations:** St Georges University Medical Center Lebanon, Beirut, Achrafieh, St Georges Street, Lebanon

**Keywords:** Shoulder, Rotator cuff, Biomechanics, Glenoid, Magnetic resonance images, Arthroscopy, Case series

## Abstract

**Objective:**

This study aims to investigate the presence of any differences between the normal population and patients with rotator cuff tears in term of glenoid morphological parameters.

**Method:**

For the purpose of this study, 82 shoulder MRIs were evaluated. The individuals included in this study were divided into the patient group (cuff tear group, n = 41) and the control group (control group, n = 41) with respect to the inclusion and exclusion criteria. For the statistical analysis, SPSS software was used.

**Results:**

Statistically significant difference was found between the patient group and the control group in terms of Glenoid Version Angle (GVA), Glenoid Axial Width (GAW), Glenoid Axial Depth (GAD), Glenoid Coronal Depth (GCD), and the Glenoid Inclination Angle (GIA). Age was found to have a positive linear correlation with the glenoid coronal height. Moreover, statistically significant difference was found between the males and females, between males with cuff tears and control males, and females with cuff tears and control females in terms of glenoid parameters. When gender was a covariate, statistically significant differences in glenoid parameters was still present between cuff tears patients and individuals with control shoulders.

**Conclusion:**

The results attained in this study are suggest that the GVA, GAW, GAD, GCD, and GIA in rotator cuff tear patients are different than those found in control patients. These measurements may be helpful in reaching the diagnosis in cases where the clinical diagnosis is equivocal. Moreover, these measurements may be helpful in planning tears management. The influence of gender on glenoid parameters and subsequent impact on shoulder pathology may help to better understand the biomechanism of rotator cuff tears and reach an individualized management for better patient care.

## Highlights

• The radiographic measurements of the glenoid parameters may be helpful when the clinical diagnosis is difficult to attain.
• These measurements can help in planning the management of cuff tears, especially in cases in which tears are resistant to treatment.
• The importance of these findings is using an individualized approach in the management of the rotator cuff tears that can be based on gender, age, and the biomechanics of the patient.

## 1 Introduction

The glenohumeral joint is a highly mobile joint with increased range of motion, which explains its anatomical predisposition for instability. Hence, the rotator cuff creates a pivot point upon which the deltoid muscle can act for further elevation of the arm. Moreover, the rotator cuff muscles maintain the depression of the humeral head into the shallow glenoid fossa during movement of the upper extremity [[1], [2], [3]]. Rotator cuff injuries is a common pathology among the adult population as it is responsible for more than 70% of all doctor visits related to shoulder complaints; and thus, remains the most common cause of shoulder pain [4,5]. The incidence of rotator cuff tears increases with age, with an incidence ranging from 20 to 54% among individuals between 60 and 80 years of age [6,7]. Patients with cuff tears have a decreased quality of life and a deteriorated functionality. Moreover, cuff tears lead to a significant economic burden as there is an increase in the utilization of healthcare resources which ranges from imaging modalities, and specialist consultations, to surgeries and subsequent physical therapy [8,9]. Rotator cuff tears are usually multifactorial and can be the resultant of a traumatic or a degenerative etiology [3]. Neer et al. postulated an extrinsic etiology for the development of rotator cuff pathologies since the anterior part of the rotator cuff muscles will be compressed against the coracoacromial arch during forward elevation of the upper extremity [3,10]. Neer’s hypothesis of rotator cuff tearing secondary to impingement was further fortified with Bigliani’s description of the acromial morphological influence on cuff tears. Bigliani et al. classified a flat acromion into type I, a curved acromion into type II, and a hooked acromion into type III. Bigliani et al., and later other authors, found a correlation between type III acromion (hooked morphology) and increased risk for rotator cuff tearing [3,11].

The literature describes modifiable and non-modifiable predisposing factors for the progression of partial-thickness to full-thickness rotator cuff tears. Some of the non-modifiable factors are anatomical factors such as glenohumeral instability, coracoid and/or acromion anatomy [2,12,13]. Hence, the literature contains the description of several factors that may be involved in the development of cuff tears. Some authors focused on certain morphological features of the glenoid, such as the glenoid inclination angle and the glenoid version angle, with an attempt to find a certain correlation with an increased risk for rotator cuff tears [[14], [15], [16]]. However, as per our knowledge, little focus has been given in the literature regarding other glenoid morphological features and parameters that may be associated with increasing the incidence of rotator cuff tears. In this study, we investigate whether there is any correlation between glenoid morphological parameters (glenoid inclination angle, glenoid version angle, glenoid coronal height, glenoid axial width, axial glenoid depth, coronal glenoid depth, and average glenoid depth) and rotator cuff tears among the Lebanese population. Moreover, we will investigate the influence of age and gender on the glenoid morphological parameters and any subsequent influence on rotator cuff injuries. To note, this article has been reported in line with the PROCESS criteria [17].

## 2 Population and methods

### 2.1 Sampling

After obtaining the approval of the institutional review board (IRB) and patient consent, retrospective analysis of the magnetic resonance (MR) images of a total of 82 individuals who presented at Saint George Hospital University Medical Center between February 2018 and June 2019 was performed for the purpose of this study.

The sample size is 82 individuals divided equally between those who underwent arthroscopic rotator cuff repair (patient group/RCT group) and those who had no findings of cuff tears as per MR images (control group). The sample size of the patient group was reached based on the number of individuals who presented for rotator cuff repair at Saint George Hospital University Medical Center between February 2018 and June 2019 with respect to the inclusion and exclusion criteria. Consequently, the sample size was not reached based on the general population. The control group was selected at random based on MR records of patients with healthy shoulders.

### 2.2 Inclusion criteria

The inclusion criteria for the patient group were met if the patient is presenting for arthroscopic rotator cuff repair after being evaluated via physical exam and MR images (MRI) giving that the MRIs were done at Saint George Hospital University Medical Center.

The number of patients who underwent an arthroscopic repair for their rotator cuff tears was 225 patients. A total of 53 patients met the inclusion criteria.

The inclusion criteria for the control group were met if the individual had a shoulder MRI done at Saint George Hospital University Medical Center.

### 2.3 Exclusion criteria

Any individual with previous or current shoulder pathology was excluded from the study. Additionally, any individual that underwent previous shoulder surgery was also excluded from the study. Moreover, any patient presenting with rotator cuff tear secondary to a traumatic event was excluded from this study.

A total of 12 patients were excluded thus reducing the number of patients in RCT group to 41 patients.

For the control group, 97 individuals met the inclusion and exclusion criteria from which 41 individuals were randomly selected.

### 2.4 Statistical analysis

Statistical analysis was performed using SPSS software (version 20; SPSS, Chicago). Data were analyzed using descriptive statistics (mean, standard deviation, median, frequency, percentage minimum and maximum).

### 2.5 Measurements

#### 2.5.1 Glenoid version angle (GVA)

**Fig. 1:**
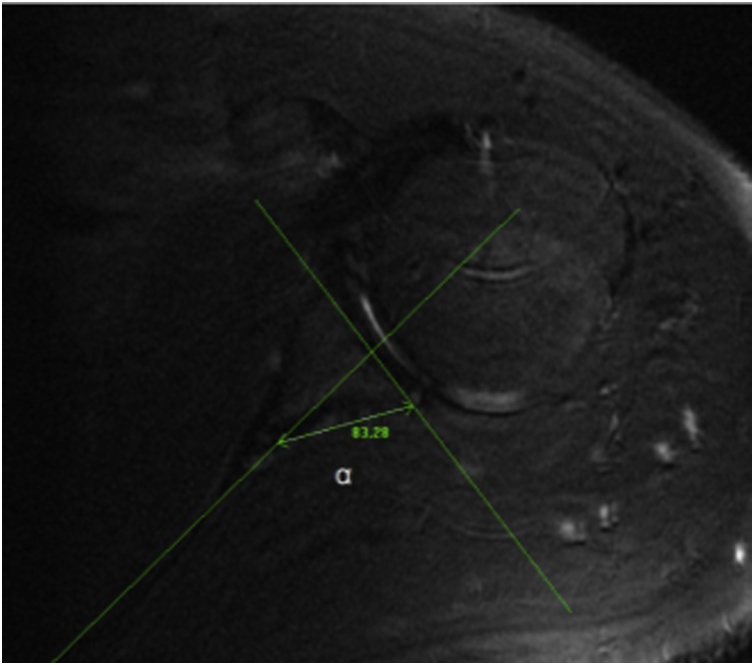
Measurement of GVA on axial MRI.

Glenoid version angle on axial MR images measured by first drawing a line through the glenoid surface’s axis on images where the posterior border of the glenoid neck was visible. Then, they drew a second line connecting the posterior glenoid neck and the point of junction of the scapular spine and scapular body medially. The glenoid version angle was calculated by the subtraction of 90 from the “a” angle with the “a” angle being the angle in the posterior medial quadrant. Moreover, a positive value for the glenoid version angle indicated an anteverted glenoid while a negative value indicated a retroverted glenoid. GVA = a − 90° (Fig. 1).

#### 2.5.2 Glenoid inclination angle (GIA)

**Fig. 2:**
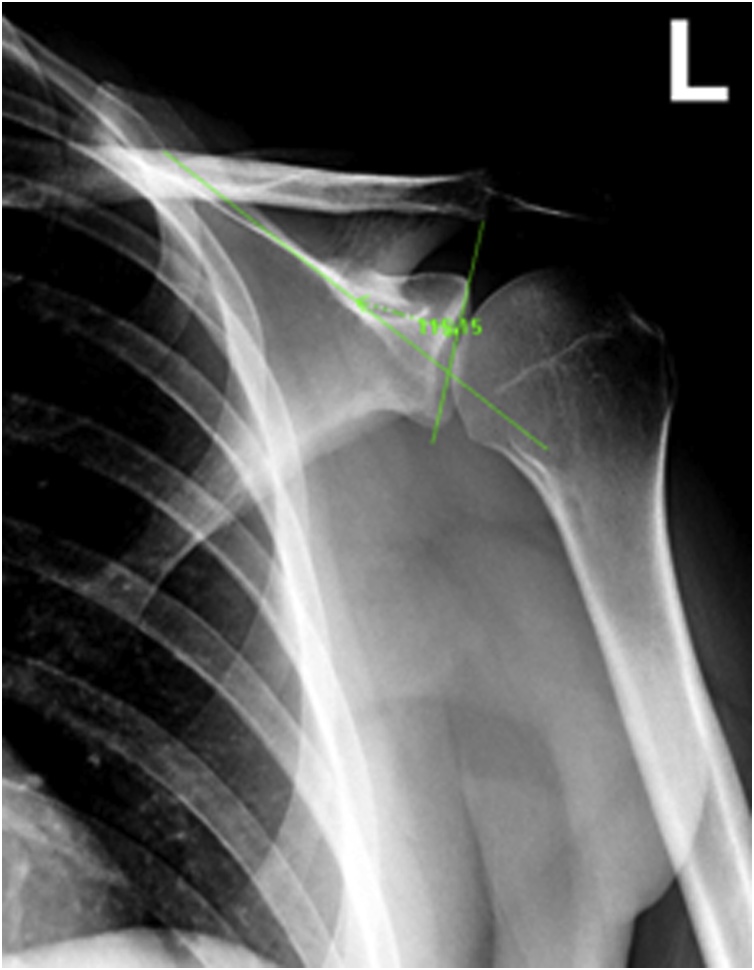
Measurement of the GIA shoulder X-ray in the anteroposterior view. GIA is the angle in the superolateral quadrant.

On axial view, the first line is drawn through the floor of the supraspinatus fossa while the second line is a line connecting the uppermost and the lowermost points of the glenoid. The glenoid inclination angle is the angle in the superolateral quadrant formed by these two lines (Fig. 2).

**Fig. 3:**
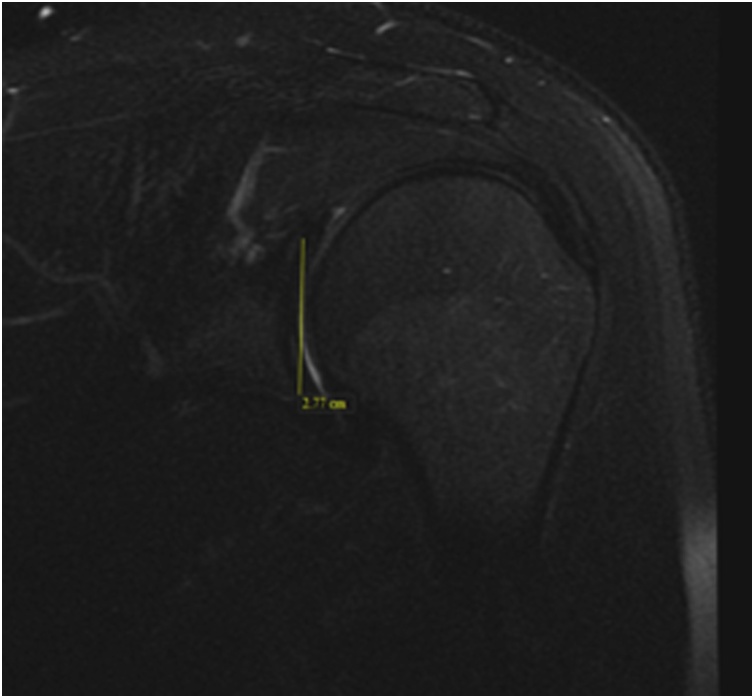
Glenoid height in the coronal view on shoulder MRI.

Glenoid Coronal Height (GCH) (Fig. 3).

**Fig. 4:**
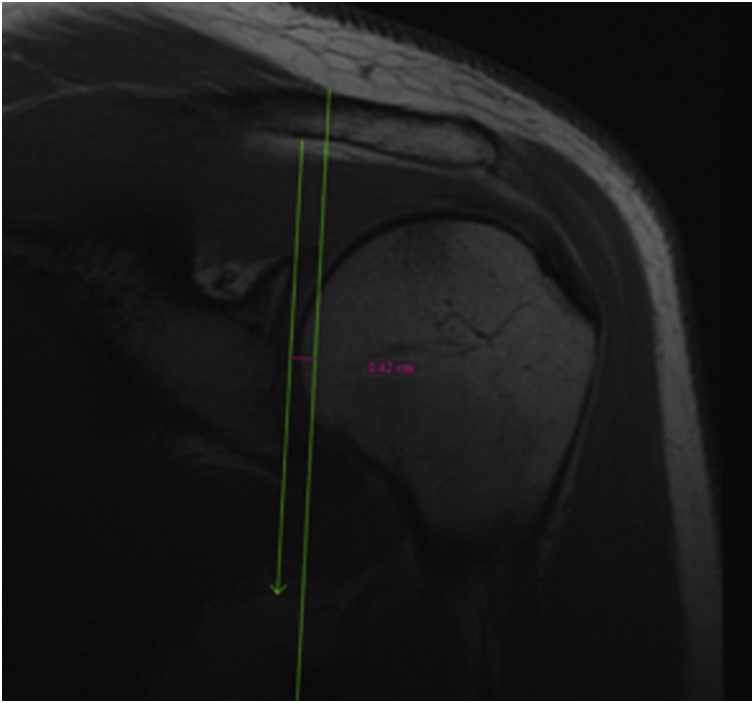
Glenoid depth as seen on the coronal view on shoulder MRI.

Glenoid Coronal Depth (GCD) (Fig. 4).

**Fig. 5:**
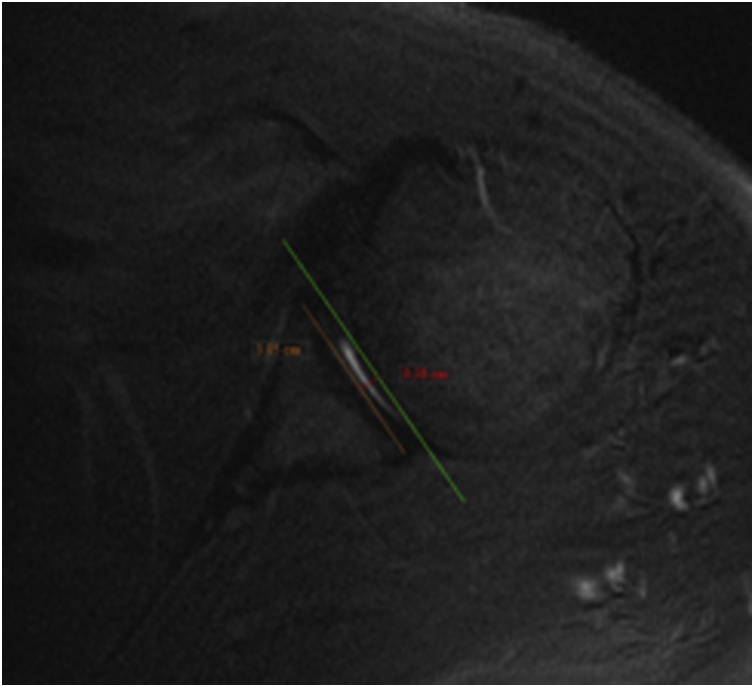
Glenoid axial width and depth.

Glenoid Axial Width and Depth (GAW and GAD) (Fig. 5).

Glenoid depth (GD) is the average of GCD and GAD.

## 3 Results

The MRIs of the 82 individuals were analyzed and included in this study. The demographic difference is shown in Table 1.

**Table 1:** The distribution of descriptive characteristics (gender and age) of the patient and control groups.

Groups		Patient group (n = 41)	Control group (n = 41)	Total
Gender	**Female**	11 (27%)	16 (39%)	27 (33%)
	**Male**	30 (73%)	25 (61%)	55 (67%)
Age (years)	**Mean ± SD**	46.54 ± 15.84	38.37 ± 13.69	42.45 ± 15.27
	**Range**	19–73	17–76	17–76

### 3.1 Comparison between the two groups for glenoid morphological parameters

For the comparison of the glenoid morphological parameters according to the two groups (patient group vs control group), the *t*-test was used for the comparison of means to assess whether a statistical significance is present. For the interpretation, Sig or P-value (Degree of significance) with α (error ratio = 5% i.e. 0.05) were compared. As such, if P-value > α → the difference is considered statistically insignificant and vice versa.

The independent *t*-test assumes the variances of the two groups being testing are equal in the given population. If variances are unequal, the Type I error rate can be affected. The assumption of homogeneity of variance can be studied using Levene's Test of Equality of Variances. This test for homogeneity of variance delivers an *F*-statistic and a significance value (*p*-value), if it is greater than 0.05 (i.e., *p* > .05), the group variances can be treated as equal, then the P-value can be used for *t*-test for Equality of Means where the equal variances is assumed. However, if *p* < 0.05, then variances are unequal. Hence, the P-value is used for *t*-test for Equality of Means where the equal variances is not assumed.

Statistical significance was found between the patient group and the control group in terms of GVA, GAW, GAD, GCD, and GIA. However, the differences were not statistically significant in GCH and GD between the patient and control groups (Table 2).

**Table 2:** The comparison of glenohumeral morphological parameters of two groups.

Parameters	Group	Group Statistics			Levene's Test for Equality of Variances			*t* test for Equality of Means	
		N	Mean	SD	Variances	F	P-value	t	P-value
GVA	Patient group	41	2.18	8.54	EVA	0.355	0.553	−1.996	0.049*
	Control group	41	5.85	8.11	EVNA			−1.996	0.049
GCH	Patient group	41	3.05	0.42	EVA	6.144	0.015	1.780	0.079
	Control group	41	2.91	0.30	EVNA			1.780	0.079
GAW	Patient group	41	2.97	0.30	EVA	2.117	0.150	−2.974	0.004**
	Control group	41	3.19	0.37	EVNA			−2.974	0.004**
GAD	Patient group	41	0.38	0.09	EVA	2.187	0.143	−3.285	0.002**
	Control group	41	0.45	0.11	EVNA			−3.285	0.002**
GCD	Patient group	41	0.42	0.08	EVA	1.652	0.202	5.980	0.000**
	Control group	41	0.32	0.07	EVNA			5.980	0.000**
GD	Patient group	41	0.40	0.05	EVA	6.034	0.016	1.099	0.275
	Control group	41	0.38	0.07	EVNA			1.099	0.275
GIA	Patient group	41	92.88	3.01	EVA	14.100	0.000	7.207	0.000**
	Control group	41	89.30	1.04	EVNA			7.207	0.000**

NB. EVA = Equal variances assumed, EVNA = Equal variances not assumed.

*Significant at level 0.05, **Significant at level 0.01.

### 3.2 Multivariate analysis

One-way multivariate analysis of variance (one-way MANOVA) (Table 3).

**Table 3:** Multivariate tests.

Multivariate Tests						
Effect		Value	F	Hypothesis df	Error df	P-value
Intercept	Pillai's Trace	1.000	27951.104	6.000	75.000	0.000**
	Wilks' Lambda	0.000	27951.104	6.000	75.000	0.000**
	Hotelling's Trace	2236.088	27951.104	6.000	75.000	0.000**
	Roy's Largest Root	2236.088	27951.104	6.000	75.000	0.000**
Group	Pillai's Trace	0.594	18.315	6.000	75.000	0.000**
	Wilks' Lambda	0.406	18.315	6.000	75.000	0.000**
	Hotelling's Trace	1.465	18.315	6.000	75.000	0.000**
	Roy's Largest Root	1.465	18.315	6.000	75.000	0.000**

**Significant at level 0.01.

To determine how the glenoid parameters (the dependent variables) differ for the patient group and the control group (the independent variable), we look at the Tests of Between-Subjects Effects table (Wilks’ Lambda row in the “Group” section) (Table 4).

**Table 4:** Tests of Between-Subjects Effects.

Tests of Between-Subjects Effects						
Source	Dependent Variable	Type III Sum of Squares	df	Mean Square	F	P-value
Group	GVA	276.186	1	276.186	3.983	0.049*
	GCH	0.423	1	0.423	3.170	0.079
	GAW	1.012	1	1.012	8.845	0.004**
	Axial GD	0.100	1	0.100	10.791	0.002**
	Coronal GD	0.202	1	0.202	35.766	0.000**
	GD	0.004	1	0.004	1.207	0.275
	GIA	263.775	1	263.775	51.942	0.000**

*Significant at level 0.05, **Significant at level 0.01.

As seen in Table 4, Table 5, the type of groups has a statistically significant effect at the same time on GVA (*F* (1, 80) = 3.983; *p* < 0.05), GAW (*F* (1, 80) = 8.845; *p* < 0.01), GAD (*F* (1, 80) = 10.791; *p* < 0.01), GCD (*F* (1, 80) = 35.766; *p* < 0.01) and GIA (*F* (1, 80) = 51.942; *p* < 0.01).

**Table 5:** Correlation between glenoid parameters and age in general and age in the two groups.

Group		Total (n = 82)		Patient group (n = 41)		Control group (n = 41)	
		Pearson Correlation	P-value	Pearson Correlation	P-value	Pearson Correlation	P-value
Glenoid parameters	GVA	0.166	0.136	0.252	0.113	0.224	0.158
	GCH	0.208	0.061	0.326	0.037*	−0.098	0.540
	GAW	−0.117	0.296	−0.018	0.913	−0.052	0.745
	GAD	−0.124	0.266	0.114	0.479	−0.175	0.274
	GCD	0.251	0.023*	0.243	0.126	−0.034	0.835
	GD	0.081	0.467	0.283	0.073	−0.152	0.342
	GIA	0.176	0.113	−0.008	0.960	0.074	0.647

*Significant at level 0.05.

### 3.3 Correlation between age and glenoid parameters

Pearson correlation was used to investigate the presence of any correlation between age in general and glenoid parameters, between age of the in individuals in the patient and control groups and the glenoid parameters (Table 5).

Statistically significant positive correlation was also found between the age of patients with rotator cuff tears (patient group) and GCH. However, no correlation was found between age in the control group and any of the glenoid parameters.

### 3.4 Glenoid parameters and gender

The difference in terms of GCH and GAW is statistically significant between males and females in the group of patients with rotator cuff tears (P-values < 0.05) (Table 6).

**Table 6:** Comparison of glenoid parameters by gender among patients with rotator cuff tears (patient group).

	Group	Group Statistics			Levene's Test for Equality of Variances			*t* test for Equality of Means	
		N	Mean	SD	Variances	F	P-value	t	P-value
GVA	Female	11	3.24	4.41	EVA	6.559	0.014	0.477	0.636
	Male	30	1.79	9.66	EVNA			0.656	0.516
GCH	Female	11	2.80	0.29	EVA	2.267	0.140	−2.402	0.021*
	Male	30	3.14	0.43	EVNA			−2.857	0.008**
GAW	Female	11	2.76	0.20	EVA	2.684	0.109	−2.952	0.005**
	Male	30	3.05	0.30	EVNA			−3.554	0.001**
Axial GD	Female	11	0.39	0.08	EVA	0.064	0.802	0.664	0.511
	Male	30	0.37	0.09	EVNA			0.682	0.503
Coronal GD	Female	11	0.38	0.05	EVA	4.075	0.050	−1.872	0.069
	Male	30	0.43	0.09	EVNA			−2.412	0.022*
GD	Female	11	0.39	0.04	EVA	0.405	0.528	−0.869	0.390
	Male	30	0.40	0.06	EVNA			−0.974	0.340
GIA	Female	11	93.71	1.70	EVA	1.801	0.187	1.060	0.296
	Male	30	92.58	3.34	EVNA			1.410	0.168

*Significant at level 0.05, **Significant at level 0.01.

Statistical significance was found between males and females in terms of GCH, GAW, GCD and GD (P-values < 0.05) among the control group (Table 7).

**Table 7:** Comparison of glenoid parameters by gender among patients in the control group.

	Group	Group Statistics			Levene's Test for Equality of Variances			*t* test for Equality of Means	
		N	Mean	SD	Variances	F	P-value	T	P-value
GVA	Female	16	4.60	3.69	EVA	10.049	0.003	−0.788	0.435
	Male	25	6.66	9.96	EVNA			−0.936	0.356
GCH	Female	16	2.69	0.24	EVA	0.007	0.932	−4.505	0.000**
	Male	25	3.04	0.25	EVNA			−4.569	0.000**
GAW	Female	16	2.91	0.33	EVA	0.233	0.632	−4.850	0.000**
	Male	25	3.37	0.28	EVNA			−4.664	0.000**
Axial GD	Female	16	0.42	0.10	EVA	0.618	0.437	−1.248	0.219
	Male	25	0.46	0.11	EVNA			−1.270	0.213
Coronal GD	Female	16	0.29	0.06	EVA	0.230	0.634	−2.379	0.022*
	Male	25	0.34	0.06	EVNA			−2.378	0.024*
GD	Female	16	0.35	0.06	EVA	0.342	0.562	−2.176	0.036*
	Male	25	0.40	0.07	EVNA			−2.180	0.037*
GIA	Female	16	89.51	0.77	EVA	1.442	0.237	1.057	0.297
	Male	25	89.16	1.18	EVNA			1.157	0.254

*Significant at level 0.05, **Significant at level 0.01.

In the case of females, statistical difference between the patient group and the control group in terms of GCD and GIA (P-values < 0.05) (Table 8).

**Table 8:** Comparison of glenoid parameters between females with rotator cuff tears and females in the control group.

Parameters	Group	Group Statistics			Levene's Test for Equality of Variances			*t* test for Equality of Means	
		N	Mean	SD	Variances	F	P-value	t	P-value
GVA	Patient group	11	3.24	4.41	EVA	0.511	0.481	−0.866	0.395
	Control group	16	4.60	3.69	EVNA			−0.837	0.413
GCH	Patient group	11	2.80	0.29	EVA	1.003	0.326	1.100	0.282
	Control group	16	2.69	0.24	EVNA			1.055	0.305
GAW	Patient group	11	2.76	0.20	EVA	3.416	0.076	−1.328	0.196
	Control group	16	2.91	0.33	EVNA			−1.455	0.158
GAD	Patient group	11	0.39	0.08	EVA	0.262	0.613	−0.805	0.429
	Control group	16	0.42	0.10	EVNA			−0.835	0.412
GCD	Patient group	11	0.38	0.05	EVA	1.922	0.178	3.957	0.001**
	Control group	16	0.29	0.06	EVNA			4.171	0.000**
GD	Patient group	11	0.39	0.04	EVA	0.903	0.351	1.372	0.182
	Control group	16	0.35	0.06	EVNA			1.478	0.152
GIA	Patient group	11	93.71	1.70	EVA	5.992	0.022	8.709	0.000**
	Control group	16	89.51	0.77	EVNA			7.662	0.000**

**Significant at level 0.01.

In the case of males, statistical difference between the patient group and the control group in terms of GAW, GAD, GCD and GIA (P-values < 0.05) (Table 9).

**Table 9:** Comparison of glenoid parameters between males with rotator cuff tears and males in the control group.

Parameters	Group	Group Statistics			Levene's Test for Equality of Variances			*t* test for Equality of Means	
		N	Mean	SD	Variances	F	P-value	T	P-value
GVA	Patient group	30	1.79	9.66	EVA	0.000	0.998	−1.832	0.073
	Control group	25	6.66	9.96	EVNA			−1.827	0.074
GCH	Patient group	30	3.14	0.43	EVA	9.507	0.003	0.989	0.327
	Control group	25	3.04	0.25	EVNA			1.036	0.306
GAW	Patient group	30	3.05	0.30	EVA	0.003	0.957	−4.197	0.000**
	Control group	25	3.37	0.28	EVNA			−4.222	0.000**
GAD	Patient group	30	0.37	0.09	EVA	2.153	0.148	−3.476	0.001**
	Control group	25	0.46	0.11	EVNA			−3.409	0.001**
GCD	Patient group	30	0.43	0.09	EVA	2.964	0.091	4.472	0.000**
	Control group	25	0.34	0.06	EVNA			4.596	0.000**
GD	Patient group	30	0.40	0.06	EVA	1.904	0.173	0.080	0.937
	Control group	25	0.40	0.07	EVNA			0.079	0.938
GIA	Patient group	30	92.58	3.34	EVA	8.367	0.006	4.870	0.000**
	Control group	25	89.16	1.18	EVNA			5.234	0.000**

**Significant at level 0.01.

One-way multivariate analysis of covariance (MANCOVA) (Table 10).

**Table 10:** Multivariate analysis.

Multivariate Tests						
Effect		Value	F	Hypothesis df	Error df	P-value
Intercept	Pillai's Trace	0.994	1987.799	6.000	74.000	0.000**
	Wilks' Lambda	0.006	1987.799	6.000	74.000	0.000**
	Hotelling's Trace	161.173	1987.799	6.000	74.000	0.000**
	Roy's Largest Root	161.173	1987.799	6.000	74.000	0.000**
Group	Pillai's Trace	0.593	17.933	6.000	74.000	0.000**
	Wilks' Lambda	0.407	17.933	6.000	74.000	0.000**
	Hotelling's Trace	1.454	17.933	6.000	74.000	0.000**
	Roy's Largest Root	1.454	17.933	6.000	74.000	0.000**
Sexe	Pillai's Trace	0.415	8.741	6.000	74.000	0.000**
	Wilks' Lambda	0.585	8.741	6.000	74.000	0.000**
	Hotelling's Trace	0.709	8.741	6.000	74.000	0.000**
	Roy's Largest Root	0.709	8.741	6.000	74.000	0.000**

**Significant at level 0.01.

In order to determine how the glenoid parameters (the dependent variables) differ for the patient group and the control group (the independent variable), we look at the Tests of Between-Subjects Effects table (Wilks’ Lambda row in the “Group” section).

As per Table 11, the type of groups (patient group vs control group) has a statistically significant effect at the same time on GVA (*F* (1, 80) = 3.991; *p* < 0.05), GAW (*F* (1, 80) = 17.594; *p* < 0.01), Axial GD (*F* (1, 80) = 11.042; *p* < 0.01), Coronal GD (*F* (1, 80) = 33.963; *p* < 0.01) and GIA (*F* (1, 80) = 54.024; *p* < 0.01).

**Table 11:** Tests of Between-Subjects Effects.

Tests of Between-Subjects Effects						
Source	Dependent Variable	Type III Sum of Squares	Df	Mean Square	F	P-value
Group	GVA	280.095	1	280.095	3.991	0.049*
	GCH	0.207	1	0.207	1.917	0.170
	GAW	1.457	1	1.457	17.594	0.000**
	Axial GD	0.103	1	0.103	11.042	0.001**
	Coronal GD	0.175	1	0.175	33.963	0.000**
	GD	0.002	1	0.002	0.673	0.414
	GIA	271.840	1	271.840	54.024	0.000**

*Significant at level 0.05, **Significant at level 0.01.

## 4 Discussion

Shoulder pain is the second most common chief complaint leading to clinic visits. Rotator cuff pathologies remain one of the most common causes of shoulder pain and dysfunctionality leading to a decrease in the quality of life and increased healthcare resources utilization [2,12,18,19]. As per our knowledge, the literature contains few reports focusing on the implications of the glenoid morphological parameters on rotator cuff pathologies. Some authors maintained that glenoid version is a risk factor for the development of rotator cuff injuries due to increased stress on the cuffs since the glenoid version influences the distribution of forces [1,20]. In their study conducted in 2007, Tokgoz et al. reported that the mean GVA was −7.1° in patients with rotator cuff tears (specifically supraspinatus tendon tears) while the mean GVA was −4.8° in the control group [20]. Moreover, Tètreault et al. (2004) established a correlation between the glenoid version and the area of rotator cuff tears. However, their study did not include a control group to which they could compare their results [1]. Dogan et al. excluded, from their study, subjects with risk factors for the development of rotator cuff tears to evaluate the influence of the glenoid axis on cuff tears and found that there was no statistically significant difference in glenoid version angle between the control group and patients with rotator cuff tears [16]. In the current study, statistically significant difference in terms of GVA was found between patients with cuff tears and control patients with lower GVA being associated with cuff tears. This might be related to the three-dimensional orientation of a glenoid with a relatively low GVA in which additional pressure is imposed on the rotator cuffs for further stabilization of the glenohumeral joint since the main function of the cuff muscles is to provide stability for the shoulder joint [21,22].

In 2003, Hughes et al. hypothesized that there might be a correlation between glenoid inclination and rotator cuff tears. The rationale behind their hypothesis was that the supraspinatus tendon may be impinged between the humeral head and the acromion since the former has a tendency to migrate superiorly. The glenoid migration depends on gravity and on the muscular forces acting on it. There should be no humeral head migration if the net force’s direction is into the glenoid. The superior shear component of this net force acting on the humeral head is supposed to increase as the angle of the glenoid inclination increases [23]. Henceforth, the deltoid muscle would require less force to cause migration of the superior humeral head when the shoulder has a greater glenoid inclination angle. Consequently, the glenoid inclination may be involved in rotator cuff pathologies such as tendon degeneration since the described superior migration causes compression on the supraspinatus tendon. Hughes et al. (2003) found, based on cadaveric subjects, that the mean difference of glenoid inclination angles between shoulders with rotator cuff tears and normal shoulders was 7.6° which is consistent with the aforementioned postulated hypothesis. The difference was statistically significant though they could not prove causality. When the glenoid articular surface has a shallow depth, the humeral head can translate easier from the glenoid, despite the potential presence of a compressive load [23]. Moreover, Tètreault et al. (2004) reported a mean difference of 10° in glenoid inclination angles between normal shoulders and shoulders with rotator cuff tears [1]. On the other hand, Kandemir et al. (2006) did not find any statistically significant difference in glenoid inclination angles between specimens with full-thickness rotator cuff tears and specimens with intact shoulders based on cadaveric studies [24]. Moreover, Bishop et al. (2009) reported that shoulders with rotator cuff tears had a lower glenoid inclination angle than normal intact shoulders (90.7° vs 92.3°) [25]. However, the difference in GIA was statistically significant between the patient group and the control group in the current study with individuals with rotator cuff tears having a higher GIA. The higher GIA leads to a decrease in the force required by the deltoid muscle to cause humeral head migration and cause rotator cuff pathology, compressive in nature. Moreover, Taniguchi et al. (2017) described the association of the humeral head translation scale on the success of massive rotator cuff tears repair [26]. As such, the literature contains conflicting results and further studies should be conducted.

Malkoc et al. (2016) reported that rotator cuff tendinitis such as partial cuff tears was associated with high glenoid coronal depth and low glenoid axial depth; and these results may be used in the diagnosis of rotator cuff tendinitis [27]. This comes in accord with our results with shows statistical significance between the patient group and the control group in terms of glenoid coronal dept and axial depth. Our results showed that higher GCH and lower GAD were associated with rotator cuff tears. The anatomical morphology dictated by a high GCD and a low GAD may reduce the stability of shoulder joint which increases the force required for the rotator cuff muscles to counterbalance this deficit. The increased workload of the rotator cuff muscles for the stabilization of the glenohumeral joint may increase the risk of a tendon tear. However, no statistical difference was found in the current study in terms of glenoid depth between the two groups.

Saygi et al. (2018) reported that the difference was statistically significant in glenoid axial width and glenoid coronal height between the group of patients with rotator cuff tears and the control group [2]. The results of the present study showed that the difference was statistically significant for the GAW but not for GCH between rotator cuff patients and the control group. In the current study, a lower GAW was found in the group of patients with cuff tears. This is likely due to increased deformation force on the rotator cuff tendons by the shape of the glenoid. Assuming the glenoid is a part of a hollow sphere, a lower GAW leads to a decrease in the volume of that sphere in which the humeral head is to fit. Subsequently, additional pressure is put on the cuff group to stabilize the shoulder joint which may increase the risk of rotator cuff pathology, degenerative in nature.

Pearson correlation was used to explore the presence of any correlation between the glenoid parameters and age in each group. Our results showed a statistically significant positive linear correlation between the age of patients with cuff tears and GCH. However, as previously stated, our results showed no association between GCH and rotator cuff tears. Moreover, no correlation was present between the age of individuals in the control group and any of the glenoid parameters. As per the literature, increasing age is associated with increased incidence of rotator cuff tears; usually degenerative in nature [28,29].

The literature does not provide insight on the influence of gender on glenoid parameters, and its association with cuff tears, or postoperative outcomes [30,31]. Our analysis of glenoid parameters within each of the two genders showed statistically significant difference between males and females in terms of GCD and GIA. Furthermore, in the male group, we found statistically significant difference in GAW and GAD. This is likely due to the male phenotype which entails a larger musculoskeletal system. Upon comparison of the glenoid parameters of males in the patient group and the control group, statistically significant difference was found between males with cuff tears and males in the control group in terms of GAW, and GAD, both of which were lower in the cuff group; GCD, and GIA, both of which were higher in the cuff group. For the female gender, there was statistically significant difference between females with cuff tears and females in the control group in terms of GCD and GIA, both of which were higher in the cuff group.

The one-way multivariate analysis of covariance (MANCOVA) was used to determine whether there are any differences between the independent groups (patient group vs control group) on more than one continuous dependent variable where the gender is the covariate and it’s linearly related to the dependent variables (the glenoid morphological parameters) and its inclusion into the analysis can increase the ability to detect differences between groups of an independent variable. The Multivariate Test (MANCOVA) analysis concluded that the effects of type of groups on the glenoid morphological parameters are still significant, even after controlling this relationship by gender. The differences in the musculoskeletal anatomy between males and females may explain the gender predilection of certain musculoskeletal pathologies.

## 5 Conclusion

The results obtained from the current study suggest that the glenoid version angle, glenoid axial width, glenoid coronal depth, glenoid axial depth, and the glenoid inclination angle in patients with rotator cuff tears are different from those in the control group. These radiographic measurements of the glenoid parameters may be helpful when the clinical diagnosis is difficult to attain. Moreover, these measurements can help in planning the management of cuff tears, especially in cases in which tears are resistant to treatment.

Age was found to be positively and linearly correlated with the glenoid coronal height; despite GCH not being associated with cuff tears. Glenoid parameters differed between males and females, between males with cuff tears and males in the control group, and between females with cuff tears and females without cuff tears. Moreover, even when gender was the covariate, significant differences in glenoid parameters was still present between cuff tears patients and control patients. The importance of these findings is using an individualized approach in the management of the rotator cuff tears that can be based on gender, age, and the biomechanics of the patient. The authors encourage the conduction of further studies, preferably prospective in nature, for better understanding of the shoulder biomechanics and their impact on shoulder pathologies; with subsequent implications on postoperative patient care, and perhaps prophylaxis against rotator cuff tears.

## 6 Limitations

This study had some limitations. First of all, measurements taken from the MRI imaging were done once and by one radiologist at our institution. The second limitation is that MRI was used in order to study bony morphology, as our study is retrospective, and most patients are evaluated mainly for shoulder pathologies with MRI imaging to study soft tissue problems as well.

## Sources of funding

No funds were received in support of this study.

## Ethical approval

Ethics committee has given approval for publication and patient consent has been obtained.

## Consent

Written informed consent was obtained from the patients for publication of this case report and accompanying images. A copy of the written consent is available for review by the Editor-in-Chief of this journal on request.

No identity identifiers are present whatsoever in the manuscript.

## Author contribution

Joseph Maalouly: contributed to the writing and editing of this article.

Antonios Tawk: contributed to the writing and referencing of this article.

Dany Aouad: contributed to the writing of this article and the submission process.

Hicham Abdel Nour: contributed to the editing of the figures and of the text.

Ghadi Abboud: contributed to the radiological images, and editing of the final text.

Elias Saidy: Contributed with data collection and editing of the article.

Georges El Rassi: contributed with the cases, writing and editing of the article.

## Registration of research studies

UIN: researchregistry5352.

## Guarantor

Dr Georges El Rassi.

## Provenance and peer review

Not commissioned, externally peer-reviewed.

## Declaration of Competing Interest

The authors declare no conflict of interest regarding the publication of this article.
